# Signal-Induced Inhibition of Telomerase Activity in HL60 Cancer Cells by Signal Transduction Using the Biophysically Activated Regulative Molecule 31 (RM31): A Pilot Study

**DOI:** 10.7759/cureus.22962

**Published:** 2022-03-08

**Authors:** Claudia B Klein

**Affiliations:** 1 Epigenetics, YENO Evolution GmbH, Vienna, AUT

**Keywords:** cell nucleus stimulation, biophysical activation, signal transduction, htert, telomerase, cancer cells

## Abstract

In this pilot study, we report the use of a novel, patented biophysical technology, which enables intranuclear access and cell nucleus stimulation, via the signal of the biophysically activated regulative molecule 31 (RM31). RM31 is the name of an isolated natural molecule found in the human body and is involved in many cellular mechanisms. We used a specific low electromagnetic field frequency to activate the RM31 molecule, which leads to specific signal transduction, to investigate the effect of telomerase activity in HL60 cancer cells. Our results revealed a dramatic inhibition in telomerase activity, a 99.5% decrease within 72 hours, with avoidance of subsequent reactivation, due to the simultaneous inhibition of human telomerase reverse transcriptase (hTERT).

## Introduction

Cancer develops when normal cells change into tumor cells. Usually, this occurs via a multi-stage process that progresses from a pre-cancerous lesion to a malignant tumor [1]. These changes are the result of the interaction between a person's epigenetic factors and three categories of external agents, including physical, chemical, or biological carcinogens. As a person ages, the incidence of cancer rises dramatically, as the overall risk accumulation is combined with the tendency for cellular repair mechanisms to be less effective. Smoking, drinking alcohol, eating an unhealthy diet, physical inactivity, and air pollution are the major risk factors for developing cancer [1]. It is estimated that approximately 30-50% of all cases of cancer could be prevented by avoiding these risk factors and instead implementing existing evidence-based prevention strategies. The properties of a cell are passed on to the next generation via both genetic and epigenetic routes [2]. Genetic information is encoded in the DNA sequence while epigenetic information is defined by DNA and chromatin modifications (DNA methylation or methylation, phosphorylation, acetylation, and ubiquitination of histone cores).

The epigenetic control of genes is complex and involves several molecular signals regulated by changes in the environment and developmental status [2]. Transcription factors, non-coding RNAs (ncRNAs), DNA methylation, histone modification, and chromatin remodeling are all epigenetic signals that regulate the accessibility and expression of genes. Through cis- and trans-acting regulatory mechanisms, transcription primarily results in a self-propagating state that establishes epigenetic states through cis-acting and non-coding polycomb domains. Furthermore, epigenetic states are reinforced via histone modifications and DNA methylation [2].

In cells, the length of telomeres is maintained by telomerase or an alternative lengthening of telomeres (ALT) mechanism [3]. Decreased telomerase activity has been shown in some normal somatic cells and it is expected that some types of normal cells have low levels of ALT-like activity. It is thought that such inherited abnormalities can contribute to cancer and aging. The telomere length maintenance mechanisms are similar because activation of each is associated with immortalization [3].

The scientific work in this pilot study is based on the knowledge that molecules have specific molecular vibrations [4] or “biophysical frequencies.” Now, for the first time, due to this novel biophysical method, this mechanism can be used for epigenetic regulation through the targeted application. Molecular vibration is understood to be a periodic motion of adjacent atoms in a molecule. These oscillations occur in every molecule and can be excited by the supply of energy, for example, by the absorption of electromagnetic radiation [5].

This novel method involves the use of biophysical stimulation of these molecular vibrations, or “cell frequencies,” by transforming the molecular structure of the biophysically activated regulative molecule 31 (RM31) in a signal structure and transporting this signal into the nucleus. This is possible through the application of patented technology of the Quantum One (QO) laboratory device (YENO Evolution GmbH, Vienna, Austria), which has been developed in the last seven years. This device works using an extremely low-frequency electromagnetic field (ELF-EMF), an electromagnetic photon field that is superimposed on several simultaneous frequencies, whereby a temporary "zero magnetic field" (i.e., any influence of the Earth's usual magnetic field is removed) is induced by overlapping and interfering with short, sharp frequencies in quick succession (called needle frequencies). The electrons from the RM31 molecule and the induced photons build the base for transforming the molecular structure into a signal structure, as both are quantum objects (wave and particle properties can be observed in both). This effect is used in its entirety for biophysical stimulation and subsequent signal transduction.

The biophysical method may be used to regulate cell function through the correction of epigenetically relevant information, with a subsequent reprogramming of ribosomal protein synthesis. This method is based on the concept that targeted electrophysical stimulation by electromagnetic-zero-gravity-photon-frequency (EM0GPF) has a significant activation reaction on intranuclear enzymes such as telomerase, among others. Thus, the aim of this study was to determine the effect of the use of this new technique on telomerase activity (by quantitative telomeric repeat amplification protocol (Q-TRAP)) and human telomerase reverse transcriptase (hTERT) mRNA expression in the HL60 human promyelocytic leukemia cell line.

## Technical report

Study design

All laboratory work performed in this study, including measurement of telomerase activity, hTERT, and telomere length, was performed by Life Length, Madrid (www.lifelength.com) and included in the Life Length Study Report, 2020. At time point zero, both Q-TRAP and hTERT assays were performed on untreated HL60 cells. These cells were then separated into two groups; one control group was treated with the RM31 molecule alone and one group was treated with the RM31 molecule in conjunction with the QO device. This device uses a specific low electromagnetic field frequency to activate the RM31 molecule, which leads to specific signal transduction. Samples from both groups were then collected at three time points: 24 hours, 48 hours, and 72 hours post-treatment. The cells and supernatant were collected from all the samples and were then used to perform the telomerase activity and hTERT assays.

Telomerase activity determination

The telomeric repeat amplification protocol (TRAP) is a two-step process for analyzing telomerase activity in cell or tissue extracts. Relative telomerase activity (RTA) modified for real time can be measured by quantitative polymerase chain reaction (qPCR) analysis (Q-TRAP). This method has the advantages of high sensitivity, speed, and a high-throughput format compared to the regular TRAP assay. In this pilot study, we assessed telomerase enzyme activity in whole-cell lysates from cell cultures. These samples were appropriately purified, pelleted, and stored at −80°C for optimal preservation.

Q-TRAP involves cellular pellets being lysed for protein extraction, which are then subsequently quantified and stored. Subsequently, telomerase protein extracts were incubated with a specific oligonucleotide substrate to allow for the enzymatic addition of telomeric DNA repeats by endogenous telomerase. Following the enzymatic reaction, telomerase extension products were then amplified and quantified by real-time qPCR. Assays were performed in triplicate and results were reported as means and standard deviation (SD). Student’s t-test was performed using SPSS (IBM Corp., Armonk, NY).

Telomerase activity results

**Figure 1:**
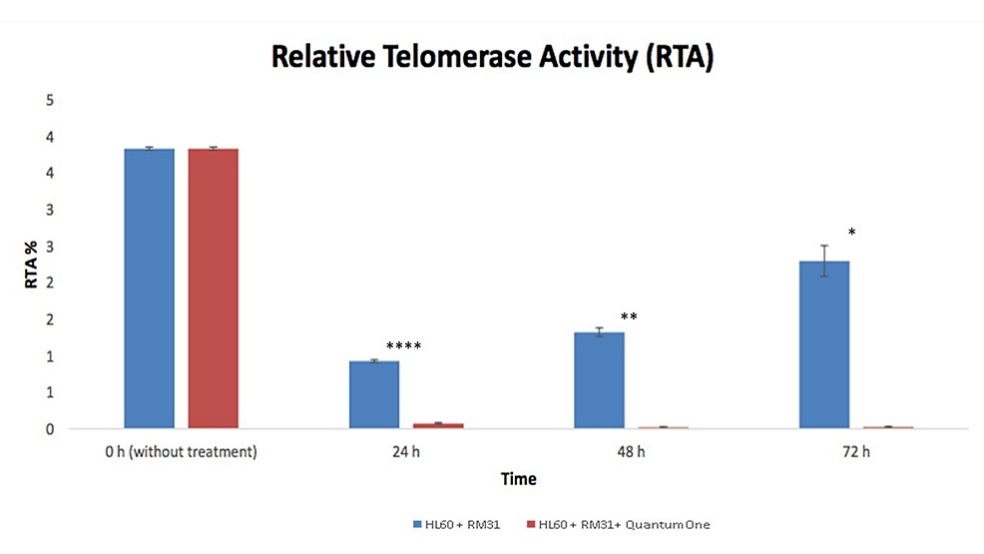
Relative telomerase activity in the HL60 + RM31 group versus the HL60 + RM31 + Quantum One group at 0 hour, 24 hours, 48 hours, and 72 hours. RM31, regulative molecule 31.

Our results showed a consistent decrease of telomerase activity: a 99.5% decrease within 72 hours after treatment with RM31 + biophysical device (QO) compared to treatment with RM31 alone (Figure 1 and Table 1). This phenomenon was observed at all time points tested (24 hours, 48 hours, and 72 hours).

**Table 1:** Relative telomerase activity at each time point. Brackets () represent % inhibition at each time point. RTA, relative telomerase activity; RM31, regulative molecule 31; QO, Quantum One.

Samples	0 hour		24 hours		48 hours		72 hours	
	RTA (%)	RTA SD	RTA (%)	RTA SD	RTA (%)	RTA SD	RTA (%)	RTA SD
HL60 + RM31	3.83 (0%)	0.02	0.93 (75.7%)	0.02	1.32 (65.5%)	0.06	2.29 (40.2%)	0.21
HL60 + RM31 + QO	3.83 (0%)	0.02	0.08 (97.9%)	0.01	0.02 (99.5%)	0.01	0.03 (99.5%)	0.01
P-values			0.00000677		0.00539		0.00951	

Determination of hTERT mRNA expression

hTERT is primarily responsible for telomere maintenance. In combination with the RNA telomerase component (hTR), dyskerin, and other small proteins, the hTERT catalyst’s function is to synthesize and lengthen telomeres.

Telomerase continuously adds a short repetitive nucleotide sequence to the ends of telomeres, compensating for the steady loss of telomere sequences at chromosomal ends that occur after each round of cell division. Upon binding of the telomere end to the catalytic site of hTERT, the hTR serves as a self-contained and unconsumed template to direct the synthesis of the telomere repeat nucleotide sequence (Life Length Study Report, 2020).

**Figure 2:**
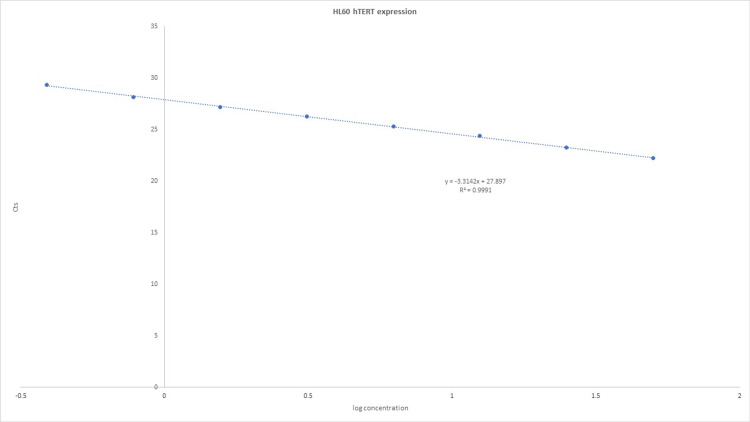
The cycle number at the threshold (Ct value) for each sample is interpolated in the curve. hTERT, human telomerase reverse transcriptase.

**Figure 3:**
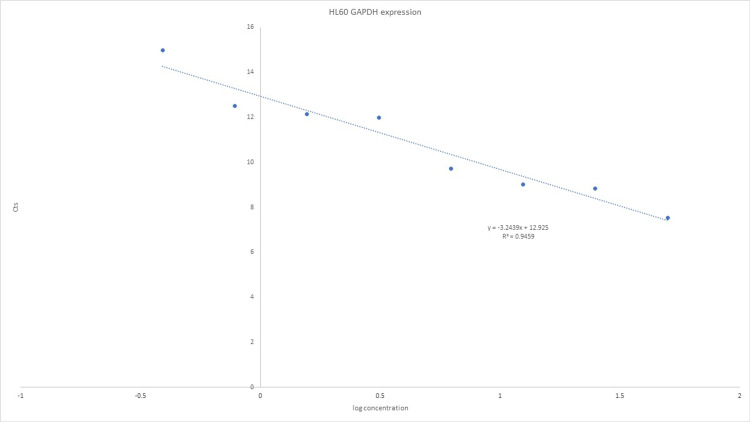
The cycle number at the threshold (Ct value) for each sample is interpolated in the curve. GAPDH, glyceraldehyde 3-phosphate dehydrogenase.

We used a real-time qPCR assay for TaqMan gene expression to detect hTERT levels (Tables 2, 3 and Figures 2, 3). RNA samples were reverse transcribed into complementary DNA (cDNA) and then pre-amplified with primers of interest to increase the signal. The TaqMan assay was then used to detect amplification. All Cts values were interpolated to a control curve generated using a series of HL60 dilutions. HL60 cells (purchased commercially from the Leibniz Institute DSMZ German Collection of Microorganisms and Cell Cultures GmbH, Braunschweig, Germany) and peripheral blood mononuclear cells (PBMCs) were used as controls. RNA was obtained using a commercially available RNA QIA-cube AllPrep RNA/DNA Mini Kit (Qiagen, Hilden, Germany). After isolation, all RNA samples were placed on ice and quantified individually using the DeNovix nanodrop (Wilmington, DE) to ensure homogeneity of samples before pooling. Assays were performed in triplicate and results were reported as means and SD.

**Table 2:** Standard curve of Ct values of RNA from HL60 cells against the log of RNA (ng) for the hTERT gene. hTERT, human telomerase reverse transcriptase.

HL60 (ng)	Log conc.	Cts
50	1.7	22.21
25	1.4	23.26
12.5	1.1	24.38
6.25	0.8	25.27
3.13	0.49	26.27
1.56	0.19	27.19
0.78	−0.11	28.15
0.39	−0.41	29.34

**Table 3:** Standard curve of Ct values of RNA from HL60 cells against the log of RNA (ng) for the GAPDH gene. GAPDH, glyceraldehyde 3-phosphate dehydrogenase.

HL60 (ng)	Log conc.	Cts
50	1.7	7.54
25	1.4	8.82
12.5	1.1	9
6.25	0.8	9.71
3.13	0.49	11.97
1.56	0.19	12.13
0.78	−0.11	12.51
0.39	−0.41	14.97

Results of hTERT mRNA expression

Our results showed that hTERT mRNA expression for the samples treated with RM31 and the QO laboratory device could no longer be determined at any of the time points (Table 4), suggesting a complete inhibition of hTERT, as glyceraldehyde 3-phosphate dehydrogenase (GAPDH) mRNA was successfully determined for these samples, indicating that the lack of a signal for hTERT was not due to a technical problem. Cts are provided as values normalized to GAPDH (average △Ct and average mean normalized expression (MNE)).

**Table 4:** RNA concentration results for samples analyzed by the hTERT assay. Measurements for the 48-hour time point were discarded due to an insufficient amount of RNA. hTERT mRNA expression and GAPDH mRNA expression are also displayed with their values normalized to GAPDH. hTERT, human telomerase reverse transcriptase; GAPDH, glyceraldehyde 3-phosphate dehydrogenase; MNE, mean normalized expression; RM31, regulative molecule 31; QO, Quantum One.

Samples	RNA (ng/µl)	RNA conc. used in assay	Cts hTERT	Cts GAPDH	△Ct	MNE
HL60 (0 hour)	410	50				
HL60 + RM31 (24 hours)	21.16	10.58	34.17	13.9	20.27	7.01E-07
HL60 + RM31 + QO (24 hours)	14.6	7.3	Undetermined	14.41		
HL60 + RM31 (48 hours)	N/A	N/A	N/A	N/A		
HL60 + RM31 + QO (48 hours)	N/A	N/A	N/A	N/A		
HL60 + RM31 (72 hours)	19.9	9.95	36.03	14.35	21.68	3.02E-07
HL60 + RM31 + QO (72 hours)	10.6	5.3	Undetermined	15.89		

## Discussion

In the European Union, the cancer burden is estimated to have risen to 2.7 million new cases (all types, excluding non-melanoma skin cancer) and 1.3 million deaths in 2020 [6]. During cancer chemotherapy, the resistance of tumor cells to antineoplastic agents is a major obstacle.

Many authors have observed that some exposure protocols to pulsed electromagnetic fields (PEMFs) can alter the efficacy of anti-cancer drugs; nevertheless, the observations are not clear [7]. Thus, tumor cells are targets of many therapeutic strategies, and tumor research is focused on searching for more efficient and specific drugs, as well as new therapeutic approaches.

Genetic and epigenetic alterations play a role in the onset and progression of cancer [8]. Epigenetics is the study of hereditable changes in gene expression without altering DNA sequences. DNA methylation, chromatin modifications, nucleosome positioning, and noncoding RNA profiles are among epigenetic changes that are reversible. It has been shown that epigenetic changes precede genetic changes and usually occur early in neoplastic development [8]. Altering epigenetic processes can cause altered gene function and cell transformation. In recent years, technological advancements have allowed us to better understand the underlying epigenetic changes that occur during carcinogenesis.

Telomeres and telomerase are currently being explored worldwide as targets for anti-cancer therapy [9]. Telomerase is a unique reverse transcriptase enzyme, considered a primary factor in almost all cancer cells, which plays a key role in regulating and reconstructing telomere length. Thus, it facilitates indefinite cell proliferation during malignancy - a hallmark of cancer. This unique characteristic is why worldwide research is currently focusing on telomerase as a potential target for drug development in cancer therapy. Deactivation of telomerase and telomere destabilization using natural products highlights a need for potential new targets for cancer therapy [9].

Telomeres are generally located at the end of chromosomes of DNA-comprising organisms [9]. Repetitive sequences of telomeric DNA, rich in guanine with a single-stranded 3′ end, fold onto the double-stranded telomere and, eventually, become a t-loop structure. This t-loop structure causes cap formation at the chromosome ends, which protects from degradation, recombination, and end-to-end fusion. Via the action of telomerase and other regulatory proteins, telomeres are usually able to maintain a certain length of the strand [9]. Thus, telomerase can be used as a biomarker due to the fact that it is missing in most somatic cells but is present in most cancer cells, making it an important target for cancer treatment [9].

Previously published studies have highlighted two mechanisms involved in telomere maintenance: the transcriptional activation of telomerase and the activation of ALT, which is a telomerase-independent telomere maintenance mechanism that used the DNA homologous recombination repair pathway. Of cancer cells, 85-95% are known to express telomerase, whereas ~5-15% exhibit activation of the ALT pathway. Although cancers with telomerase activation acquire the ability to elongate telomeres, the telomere length in prostate cancer has been found to be shorter than that of normal control tissue [10].

Okamoto and Seimiya published a study involving a well-established tumorigenesis model, in which telomeres in human somatic cells decreased in length following each cell division [10]. After 50-60 cell cycles, cells with shortened telomeres were seen to result in replicative senescence by chromosomal instability and p53 activation, thought to be induced by the DNA damage response according to telomere shortening. However, some cells that can overcome senescence continue to proliferate. As a result, they become critically short, which induces cell death. However, some of the cells that activate telomerase become immortal, which ultimately leads to cancer development [10].

In humans, suppressing telomerase activity, as well as maintaining short telomeres, is a protective mechanism against cancer [11]. Most types of primary cancer exhibit telomerase activation, which triggers uncontrolled cell proliferation. Published studies have shown that hTERT activation also affects cancer development through activities other than the canonical function of mediating telomere elongation [11].

Currently, one of the popular areas of tumor research worldwide is that of external fields. In their previously published study, Loja et al. investigated the influence of a PEMF and a hypothetic field of the pulsed vector magnetic potential (PVMP) on the growth of tumor cells, as well as the possible growth inhibition effect of the PVMP [7]. Their results showed that a PEMF of 125 Hz and 625 Hz for 24-48 hours increased proliferation activity in both the COLO-320DM and ZR-75-1 cancer cell lines used. However, in contrast, the methods employed did not confirm a significant inhibitory effect of the hypothetic PVMP field on tumor cells.

Cellular signal transduction is an important step in the processing of extracellular signals (messenger substances such as hormones and neurotransmitters), as well as internal (e.g. blood pressure) and external stimuli (e.g. vision and hearing). Important biological processes that are essentially regulated by signal transduction are gene transcription and cell proliferation. In this context, transport signals are of special importance.

The utility and targeted use of intracellular transport signals play a considerable role in specific therapeutic interventions [12]. Over the years, several research groups worldwide have identified diverse intracellular transport signals, which have greatly improved our knowledge of intracellular transport. Signals are specific information. This information transfer results in diverse biochemical reactions. Thus, the question of the importance of using the information in cells, and specifically in epigenetics, for the subsequent development of new drugs is of paramount interest. Hence, we designed this study to investigate the potential use of this technique in the development of cancer therapies.

The results of our pilot study showed a marked inhibition of telomerase activity in the HL60 cell line using the novel biophysical method, and remarkably, the inhibition was achieved solely by signal transduction of relevant intranuclear transport signals. Presumably, this results in the potential to regulate cell function by correcting epigenetically relevant information with subsequent new programming of ribosomal protein synthesis.

Carcinogenic diseases are frequently linked to defective de novo DNA methylation. We hypothesize that once the RM31 signal reaches the cell nucleus, through the binding on importin alpha, it undergoes a process of selective de novo demethylation and epigenetic regulation. This happens via an autocatalytic reaction through the enthalpy of reverse spin reactions (reverse spin flips).

A spin flip is a realignment, particularly a reversal, of the spin of an electron. In this process, an energy barrier must be overcome, due to the given magnetic field in the QO laboratory device used. These reverse spin flips release an enthalpy that can specifically overcome the binding energy of the de novo methyl group at epigenetic positions and release the bond without affecting the natural methyl groups or other molecules (which have higher binding energy). The physical calculations of low- and high-spin states form the basis for the enthalpy calculations to overcome the binding energy.

The energy induced by the signal through the biophysically activated RM31 can therefore be used for selective correction of key positions. Consequently, the resulting corrected translation leads to reprogramming of bioprotein synthesis. The de novo demethylation, as well as the hypothetically assumed reprogramming of ribosomal protein synthesis (correction of the erroneous ribosomal protein synthesis), are currently being investigated and demonstrated in a future study by our group.

## Conclusions

With current advancements in cancer detection and treatment and improving survival rates, the treatment and reduction of treatment-associated side effects are increasingly important to improve the standard of life for cancer patients, as well as optimize treatment of the disease.

The results of our pilot study showed a marked inhibition of telomerase activity in the HL60 cell line using the novel biophysical method. This new method has huge clinical potential in the development of new, highly efficient, and minimal, or zero, side effect drugs as illnesses could ideally be treated utilizing such biophysically activated substances, using only stimulated signals. This pilot study is currently being followed by a larger in vitro study of four months duration, using higher cell concentrations and more time points to verify the findings observed in the present study. Additionally, the apoptosis in the HL60 cells after different time points will also be verified.
